# Navigating a Complex Presentation: Management of Hypernatremic Dehydration, Acute Kidney Injury, Hyperkalemia, and Metabolic Acidosis in a Patient With Down Syndrome: A Case Report

**DOI:** 10.7759/cureus.46053

**Published:** 2023-09-27

**Authors:** Mahmoud AL-Nattah, Ahmad Abdullah, Nehal Alkhateeb, Hedaya Abu Qaoud, Alaeddin Al Ali, Ola Alzakeebeh

**Affiliations:** 1 Pediatrics and Neonatology, Royal Medical Services, Jordan Armed Forces, Amman, JOR; 2 Pediatric Medicine, Royal Medical Services, Jordan Armed Forces, Amman, JOR; 3 Nursing, Royal Medical Services, Jordan Armed Forces, Amman, JOR; 4 Neonatology, Royal Medical Services, Jordan Armed Forces, Amman, JOR

**Keywords:** prerenal acute kidney injury, down syndrome, hyperkalemia, metabolic acidosis, hypernatremic dehydration

## Abstract

Worldwide, gastroenteritis is a well-known cause of dehydration in pediatric patients and can be life-threatening due to subsequent electrolyte disturbance or dehydration itself. In this case, we present an infant with Down syndrome (karyotype: 21 trisomy) who presented to us with moderate hypernatremic dehydration associated with severe hyperkalemia, moderate metabolic acidosis (pH: 7.1, random blood glucose: 80-110 mg/dL), and elevated kidney function tests secondary to the gastroenteritis caused by *Entamoeba histolytica* infection. The patient is being followed up by the pediatrics genetics clinic for growth and development, with regular screening for thyroid and celiac diseases, and he has no major heart, gastrointestinal, or renal anomalies. This unique and complex presentation of electrolyte disturbance and dehydration associated with a susceptible condition of Down syndrome deserves special attention with precise management which can be challenging. We managed the patient as a case of hypernatremic dehydration with gradual correction of serum sodium and dehydration, while concurrently managing hyperkalemia by routine methods (beta agonist inhalers, insulin, dextrose 10%) with close laboratory and clinical monitoring at the pediatric intensive care unit. The pediatric nephrology team was also consulted while delineating the management plan. As the patient’s condition eventually resolved with normal kidney function and electrolytes, metabolic acidosis also resolved, with good oral intake and urine output, stable vitals, and was discharged after 72 hours. In conclusion, this case showed that pediatric patients with susceptible conditions such as Down syndrome with gastroenteritis can present with a lethal combination of dehydration and/or electrolyte disturbance, making close monitoring and prompt management paramount in such cases.

## Introduction

According to data from the World Health Organization (WHO), diarrheal diseases are the second leading cause of death in children younger than five years of age worldwide, accounting for approximately 1.7 billion cases and 525,000 deaths each year [1]. A cross-sectional study done in New Delhi, India (2015) showed that out of 6,527 samples of patients with diarrhea, *Entamoeba histolytica* was identified in 0.64% of cases [2].

Vomiting and diarrhea can commonly present with dehydration associated with normal/increased/decreased serum levels of potassium, sodium, and hydrogen ions, affected by several factors, including actual loss of electrolytes and fluid and compensatory body mechanisms (aldosterone, renal K/H pump). According to a cross-sectional study conducted among 117 children with acute gastroenteritis in a tertiary care hospital in India, isonatremia was seen in 58%, followed by hyponatremia in 35%, and hypernatremia in only 7%. Furthermore, 83% had isokalemia, 12% had hypokalemia, and 5% had hyperkalemia [3]. Another retrospective, observational, hospital-based study done in Dhahran, Nepal involving 57 children with gastroenteritis and diarrhea showed metabolic acidosis in 15 (94%) while one (6%) had metabolic alkalosis, with similar estimations regarding sodium and potassium (hyponatremia: 56%, hypernatremia: 10%, hypokalemia: 46%, hyperkalemia: 3%) [4].

Gastroenteritis is a common cause of dehydration in pediatric patients which can be associated with several electrolyte and acid-base disorders, especially if associated with previous comorbidities that can hinder normal protective physiological responses. A small study in Japan revealed that kidney function in children with Down syndrome is 80% of average Japanese children their age suggesting that children with Down syndrome have less effective kidneys due to less functioning gromeruli or smaller kidneys [5]. Another retrospective cohort study conducted in Belgium showed similar results regarding kidney size and function with an estimated glomerular filtration rate that was decreased compared to normal limits (42%), with 14% having undiagnosed renal or urinary tract anomalies which may have contributed to their kidney function status [6]. Regarding malnutrition and decreased oral intake, a questionnaire administered to 47 normal developing children and 17 children with Down syndrome showed that children with Down syndrome tend to have more feeding difficulties [7], putting them at risk for dehydration and associated electrolyte abnormalities.

As our patient presented with moderate hypernatremic dehydration associated with severe hyperkalemia and moderate metabolic acidosis, this complex presentation, along with his condition (Down syndrome), deserved to be addressed to increase awareness about similar presentations in the future.

## Case presentation

An 11-month-old male patient, a known case of trisomy 21 (Down syndrome) and previously healthy, presented to our emergency department with hypoactivity, decreased oral intake, vomiting, and diarrhea of two days duration. He vomited nearly four to five times a day, spontaneous, non-projectile vomit, with food particles and yellowish to clear fluids, devoid of blood. He had five to six bouts of foul-smelling mucoid diarrhea that was not associated with blood. The mother also reported fever with undocumented temperature. Upon clinical examination, the patient was hypoactive, ill-looking, and coughing intermittently. He had tachycardia (heart rate: 122 beats/minute), fever (temperature: 39°C), dry mucous membranes, tearless crying, sunken eyes, depressed anterior fontanel, and delayed capillary refill (three seconds). His blood pressure was 93/54 mmHg. The patient’s nutrition included mainly formula milk (five to six feedings of about 180 mL), with an average of one to two meals of table food (smashed as a soft diet), and at the time of presentation, he was consuming about half his regular intake for three days according to the mother. He weighed 7 kg and his height was 65 cm (below the fifth percentile, on the 10th percentile, respectively, according to the Centers for Disease Control and Prevention growth charts for children with Down syndrome). The patient was a product of a non-consanguineous marriage. The mother was 41 years old G5P5 (her last birth was this patient at 40 years of age), a non-smoker with no known medical conditions, and all other siblings were well and healthy. His initial laboratory results are presented in Table 1.

**Table 1:** The initial laboratory results of the patient. CBC: complete blood count; Hgb: hemoglobin; RBC: red blood cell; WBC: white blood cell; PLT: platelet; RDW: red cell distribution width; MCV: mean corpuscular volume; LFT: liver function test; AST: aspartate aminotransferase; ALT: alanine transaminase; ALP: alkaline phosphatase; TSB: total serum bilirubin; KFT & E: kidney function test and electrolytes; Cr: creatinine; BUN: blood urea nitrogen; Na: sodium; K: potassium; Cl: chloride; RBS: random blood sugar; Ca: calcium; VBG: venous blood gases; hpf: high-power field; CRP: C-reactive protein

CBC	LFT	KFT and E	VBG	Urine analysis	Stool analysis	other
Hgb: 13.9 g/dL (normal: 11–13.5 g/dL)	AST: 36 U/L (normal <40 U/L)	Cr: 2.1 mg/dL (normal 0.2–0.43 mg/dL)	PH: 7.1 (normal: 7.34–7.46)	WBC: 8–10/hpf (normal <5/hpf)	Parasite: E. histolytica trophozoites and cysts (normal: none)	Serum acetone: Negative (normal: negative)
RBC: 5.57 × 10^6^/UL (normal: 3.5–5.5 × 10^6^/UL)	ALT: 31 U/L (normal <40 U/L)	BUN: 60 mg/dL (normal: 6–20 mg/dL)	HCO_3_: 3.9 mmol/L (normal: 22–28 mmol/L)	RBC: None (normal <5/hpf)	Blood: Positive (normal: negative)	CRP: 17 mg/L (normal: <11 mg/L)
WBC: 8.9 × 10^3^/UL (normal: 6–17.5 × 10^3^/UL)	ALP: 247 IU/L (normal <350 IU/L)	Na: 160 mmol/L (normal: 135–145 mmol/L)	CO_2_: 12.6 mmHg (normal: 38–52 mmHg)	Glucose: Negative (normal: negative)	WBC: Positive (normal: negative)	
PLT: 607 × 10^3^/U/L (normal: 150–450 × 10^3^/U/L)	TSB: 0.1 mg/dL (normal: 0.3–1.0 mg/dL)	K: 6.75 mmol/L (normal 3.5–5 mmol/L)	O_2_: 64 mmHg (normal: 24–48 mmHg)	Ketone bodies: Negative (normal: negative)	Mucous: Positive (normal: negative)	
RDW: 18.7% (normal: 12.3–15.6%)		Cl: 96 mmol/L (normal: 90–110 mmol/L)				
MCV: 81.9 fL (normal: 73-85 fL)		RBS: 111 mg/dL (normal: 60–100 mg/dL)				
		Ca: 9 mg/dL (normal: 8.8–10.8)				

**Figure 1:**
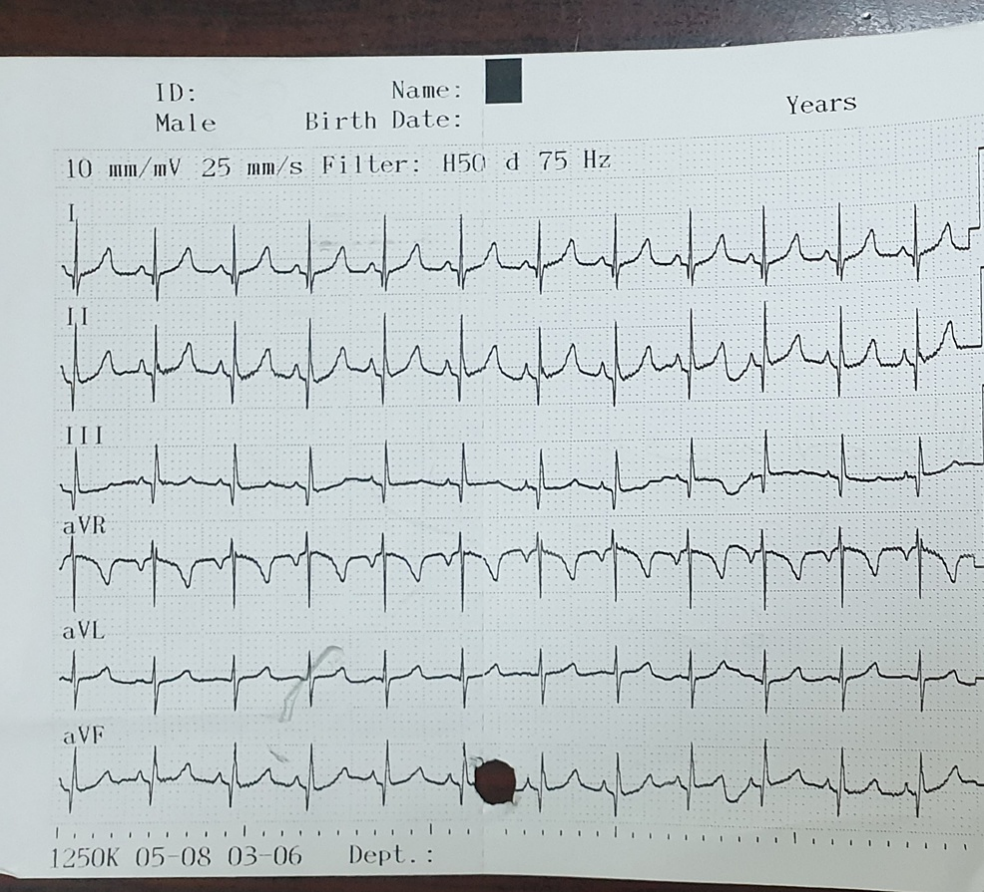
The initial ECG of the patient. Peaked T-wave in leads 1 and 2.

**Figure 2:**
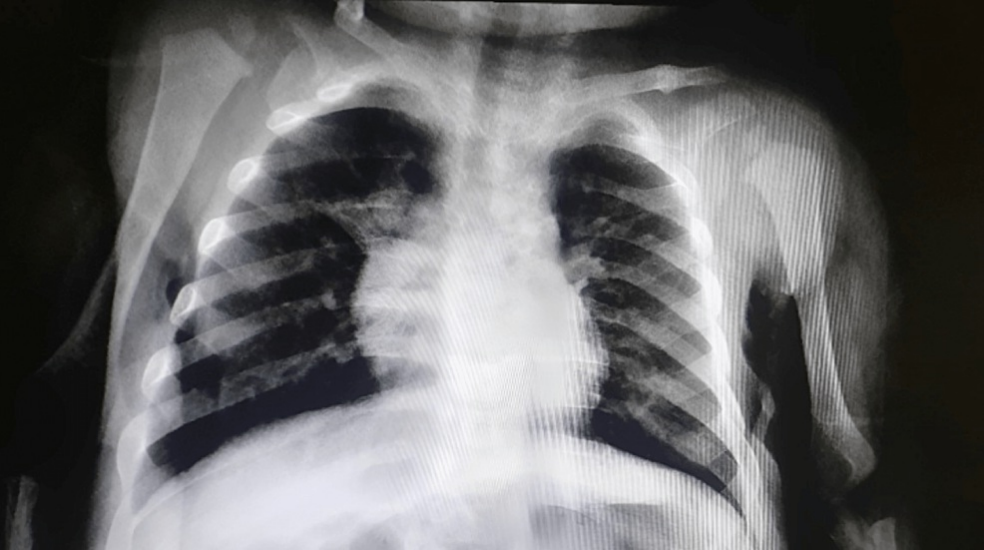
Chest X-ray of the patient. Bilateral lung infiltrations with increased vascular markings.

Urine and blood cultures were drawn which yielded negative results (no growth). An ECG and chest X-ray were ordered and the findings are shown in Figure 1 and Figure 2, respectively.

The following management plan was followed [8]: The patient was given two intravenous normal saline pushes (0.9% NaCl solution, 20 mL/kg) over 20 minutes per push. He was initially put on an NPO diet and admitted to the pediatric ICU. His vital signs were continuously observed, with continuous ECG monitoring. A urinary bladder catheter was inserted to measure urine output.

For managing and addressing hyperkalemia, continuous salbutamol nebulizers were started (3 mg diluted in 2 mL normal saline every 20 minutes), along with calcium gluconate 10% (1 mL/kg = 100 mg/kg) was given STAT over 10 minutes, NaHCO_3_ (1-2 mL/kg = 1-2 mEq/kg ) STAT over five minutes each in different IV cannula sets, dextrose 10% STAT (5 mL/kg), and rapid-acting insulin (0.1 IU/kg).

For hypernatremia and dehydration, continuous intravenous fluid was started to cover maintenance (100 mL per first 10 kg/24 hours) and moderate dehydration (10% of weight, half was added the first eight hours, and the other half was added over the next 16 hours), where the intravenous fluid concentration consisted of dextrose 10% with one-fourth normal saline (0.225% = 38 mEq/L), with continuous intravenous rapid-acting insulin (0.1 IU/kg/hour) to manage hyperkalemia as well.

The patient was started on oral metronidazole (10 mg/kg TID) to manage ameba infection, along with intravenous ceftriaxone (75 mg/kg divided q12 hours) for presumed concurrent chest infection due to the clinical and radiological presentations.

He was also followed up with glucocheck every 30 minutes for the first two hours, then every hour, with serial laboratory investigations for kidney function tests, venous/arterial blood gases, and electrolytes every four hours. Serial laboratory investigations with subsequent actions are shown in Table 2.

**Table 2:** Follow-up patient laboratory results. Cr: creatinine; BUN: blood urea nitrogen; Na: sodium; K: potassium; Cl: chloride; RBS: random blood sugar; Ca: calcium; VBG: venous blood gases

	4 hours later	8 hours later	14 hours later	20 hours later	26 hours later	32 hours later	41 hours later	52 hours later	64 hours later
Cr, mg/dL (normal: 0.2–0.43 mg/dL)	1.7 (decreased from 2.1)	1.1	0.6	0.5	0.35	0.33	0.35	0.34	0.33
BUN, mg/dL (normal: 6–20 mg/dL)	56 (decreased from 60)	43	18	15	9.8	8.3	3.9	7.59	8.3
K, mmol/L (normal: 3.5–5 mmol/L)	3.39 (decreased by 3.36)	3.4	4.68	5	4.4	4.46	4.32	4.1	4.6
Na, mmol/L (normal: 135–145 mmol/L )	151 (decreased from 160 = 2.25 per hour)	154	156	149	144	144	140	137	144
RBS, mg/dL (normal: 60–100 mg/dL)	90	100	125	62	120	80	69	84	75
Ca, mg/dL (normal: 8.8-10.8)	9	8.6	9	-	-	-	8.6	8.8	9
Cl, mmol/L (normal: 90–110 mmol/L)	99	100	104	104	105	-	-	106	-
pH (normal: 7.34–7.46)	7.18	7.16	7.17	7.17	7.19	7.29	7.24	7.3	-
HCO_3_ mmol/L (normal: 22–28 mmol/L)	8.2	9.2	12	10.7	12.4	13.5	12.2	13.5	-
O_2_,mmHg (normal in VBGs: 24–48 mmHg, normal in ABGs: 75–100 mmHg)	190 (ABGs)	114.5 (ABGs)	37.2 (VBGs)	62 (VBGs)	154 (ABGs)	110 (ABGs)	163 (ABGs)	110 (ABGs)	-
CO_2_,mmHg (normal in VBGs: 39–54 mmHg, normal in ABGs: 33–47 mmHg)	22.5 (ABGs)	26.4 (ABGs)	32.9 (VBGs)	30 (VBGs)	33 (ABGs)	35 (ABGs)	28.6 (ABGs)	27 (ABGs)	-
Urine output (normal >0.5–1.5 mL/kg/hour)	55 ml	70 mL	75 mL	88 mL	95 mL	98 mL	-	-	-
Other	-	-	Serum acetone negative	-	CRP negative	-	-	-	-
Action	D/C salbutamol and insulin Start 5% n/s Cover KCl 4 mEq/100 mL	-	G5% ½ ns 2 mEq KCl/100 mL	Start oral feeding	G5% 1/4 n/s 1 mEq KCl/100 mL. Oral feeding	D/C urinary catheter	KVO full oral intake	Transfer to the ward	Discharge at 72 hours. Follow-up after 2 days

## Discussion

Although upper respiratory tract, middle ear, and chest infections are increased in pediatric patients with Down syndrome due to immunological dysfunction that is not fully understood [9], there is no evidence that pediatric patients with Down syndrome, apart from structural anomalies, have increased gastrointestinal infections or impaired resistance to common causative viruses or parasites, and poor hand washing after toileting may contribute [10]. However, putting their limited renal capacity and decreased oral intake into consideration as mentioned above [5-7], their poor mechanisms to accommodate dehydration and electrolyte disturbance can put them at risk.

Patients with gastroenteritis can present with a variety of clinical and laboratory manifestations that can affect the initial response and management of the case. Whether to hydrate orally as an outpatient or with intravenous infusions as an inpatient depends on both types of electrolyte disturbance and the severity of dehydration. Several studies have shown a better correlation between urea (high readings) and bicarbonate levels (less than 13 mmol/L) with the severity of dehydration and need for admission and intravenous fluid replacement compared to oral replacement as an outpatient [11-13].

Hyperkalemia mainly occurs due to two factors: increased extracellular shifting or decreased renal secretion. Hyperkalemia with EKG changes or high serum levels (>6.5 mEq(mmol)/L) is considered an emergency and should be treated promptly as it may cause life-threatening cardiac arrhythmias and muscle paralysis. Treatment should be started by stabilizing cardiomyocytes with calcium gluconate given intravenously (in case the patient does not have any contraindication to calcium gluconate, such as hypercalcemia or digoxin toxicity), with potassium intracellular-shifting treatment such as beta-agonists, insulin, and sometimes diuretics to increase potassium urinary loss. In refractory cases or cases with very severe hyperkalemia, hemodialysis remains the best modality to remove potassium from the body [14]. However, gastroenteritis is not a common cause of hyperkalemia, as mentioned previously [3,4]. Potassium and sodium concentration gradients are strictly maintained across the myocardial cells by ATPase pumps that pump sodium outside and potassium inside the cell, and this plays a vital role in maintaining cardiac rhythm. Hyperkalemia implies increased extracellular potassium, which may decrease potassium gradient across the cellular membrane decreasing membrane resting potential, which, in turn, decreases activated sodium channels causing decreased inward sodium movement, resulting in decreased conduction of an impulse with prolonged depolarization and subsequent arrhythmias. The same principle applies to skeletal muscles which may manifest as paralysis [15].

Sodium is one of the major solutes contributing to osmolality and can lead to hyperosmolality with increasing levels, drawing more water by osmosis and causing cell dehydration. Regarding brain cells, rapidly occurring hypernatremia can lead to cellular shrinkage and vascular rupture with cerebral and subarachnoid hemorrhage. Fortunately for most cases, the progression of hypertonicity is slow enough to allow brain cells to adapt, minimizing the risk; however, accumulated organic osmolytes during adaptation are slow to leave brain cells, making rapid correction of extracellular hypertonicity compared to intracellular hyperosmolarity risky for brain edema. To do this safely, it is advised that the rate of correction should not exceed 12 mEq/L/day (0.5 mEq/L/hour) (1 mEq Na+ = 1 mmol Na+) [16]. Hypernatremia in the United States occurs usually in hospital settings in children who have limited access to fluids. The incidence is estimated to be no more than 1% in inpatients. Another common scenario is breastfeeding-related hypernatremia (1-2%), especially in premature newborns. As for gastroenteritis, it does not commonly contribute to hypernatremia (20%) [17]. A retrospective study was done in Bangladesh involving 1,330 children who were admitted due to gastroenteritis and diarrhea in 1979. The study showed that the incidence of hypernatremia correlated inversely with patient age, where hyponatremia was seen more in older patients. There was a good relation between malnutrition and type of dehydration. Hyponatremia was seen more in patients with malnutrition while more patients with good nutrition showed hypernatremic dehydration [18]. Hypernatremia was traditionally classified into mild (150-155 mEq/L), moderate (155-170 mEq/L), and severe (more than 170 mEq/L). Choosing the ideal fluid for correction and calculating the exact replacement fluid requirement in children with hypernatremia depends on the status of hydration and serum sodium level. Therapeutic fluids include dextrose 5% with water, 0.9% NaCl (normal saline), 0.45% NaCl (1/2 normal saline), or 0.2% NaCl (about 1/4 normal saline). Sometimes alternating between fluid types is needed where a sudden drop in tonicity carries potential neurological risks [19]. In 2005, the American Academy of Pediatrics proposed a protocol for hypernatremic dehydration correction using sodium chloride/dextrose concentrations prepared by using dextrose with calculated amounts of hypertonic saline 3% that differ from patient serum sodium levels to provide Na+ concentration around 10 mEq/L, which provides optimum correction of hypernatremia and dehydration. However, very little is known regarding the management of severe hypernatremic dehydration when associated with acute kidney injury in children [20].

Metabolic acidosis is not uncommon in children and infants presenting with dehydration, and the pathophysiology is due to several factors, including bicarbonate loss in stool or urine in some types of renal tubular acidosis, as well as glycogen depletion in starvation leading to ketosis which consumes pre-existing bicarbonate as a physiological buffer. It occurs earlier in children than adults. Poor tissue perfusion due to dehydration produces lactic acidosis consuming bicarbonate and decreased renal perfusion and glomeruler filtration limiting hydrogen ion secretion [21]. In the intensive care unit of Dhaka Hospital, a retrospective chart analysis was performed on data from children who were treated. The study showed that patients who presented with diarrhea and had metabolic acidosis had higher case-fatality rates compared with those who did not have metabolic acidosis. They were independently associated with acute renal injury and severe sepsis, underscoring the importance of monitoring children to identify these important parameters early, allowing for early intervention which can decrease mortality rates [22]. Several studies have shown that diarrheal children showed increased fatal outcomes when metabolic acidosis was associated with acute kidney injury [23,24] and dehydration [25].

## Conclusions

Regarding this case, several points should be addressed, such as the importance of close monitoring in treating dehydration and hypovolemia with gradual correction of serum sodium. Our patient was given two normal saline pushes for resuscitation and was put on dextrose 10% 1/4 normal saline for maintenance and defect (dextrose 10% was preferred due to insulin treatment for hyperkalemia). His initial serum sodium was 160 mmol(mEq)/L which decreased to 151 mmol(mEq)/L in four hours, necessitating the shift to dextrose 10% normal saline with subsequent adjustments on NaCl concentration to assure gradual correction according to sodium serum readings.

In parallel, hyperkalemia treatment was initiated concurrently along with hypernatremic dehydration management with routine methods (calcium gluconate, sodium bicarbonate, beta-2 agonists inhalers, insulin, and 10% dextrose), where the serum readings went from 6.75 mmol(mEq)/L to 3.39 mmol(mEq)/L in four hours, necessitating termination of hyperkalemia management and adding potassium maintenance to total intravenous fluids. Metabolic acidosis and acute renal injury resolved gradually with ongoing treatment for dehydration, hypernatremia, and hyperkalemia.

In conclusion, patients with Down syndrome and gastroenteritis can present with a fatal combination of electrolyte and acid/base disturbance along with dehydration. Given their limited ability to withstand these changes, it is preferable to perform routine kidney function tests with electrolytes and pH with careful clinical evaluation upon presenting with diarrhea or vomiting. Upon existing electrolyte disturbance, it is highly advisable to perform close clinical and laboratory monitoring for the patient as hydration and electrolyte statuses, with subsequent actions, can shift rapidly.
